# Environmental Governance Goals of Local Governments and Technological Innovation of Enterprises under Green Performance Assessment

**DOI:** 10.3390/ijerph20031996

**Published:** 2023-01-21

**Authors:** Xingshuai Wang, Ehsan Elahi, Zainab Khalid, Mohammad Ilyas Abro

**Affiliations:** 1 School of Economics, Shandong University of Technology, Zibo 255000, China; 2 School of Economics and Management, Southeast University, Nanjing 210096, China; 3 Department of Basic Sciences and Humanities, Dawood University of Engineering and Technology, Karachi 74800, Sindh, Pakistan

**Keywords:** green economic development, local governments, environmental governance, emission reduction targets, technological innovation

## Abstract

The current study empirically estimates the impact of local government environmental governance on enterprise technological innovation from the perspective of a green political performance assessment of local governments with Chinese characteristics. Fourteen years of data (from 2006 to 2019) on pollutant emissions, and the patents of A-share listed companies were collected from 230 cities in China. A fixed effect model and tool variable method were applied to empirically analyze the objectives of the study. The results show that the environmental governance formulated by the local government has regional differences, which are shown as lower governance indicators for underdeveloped areas and higher governance indicators for developed areas. Environmental governance has a greater promotion effect on technological innovation in enterprises in developed regions, as well as in large and private enterprises. Moreover, mechanism analysis showed that the local governments preferred the path of financial subsidies to promote the level of technological innovation in enterprises. This study provides a foundation for attaining the “win–win” scenario of local government environmental stewardship and high-quality green economic growth.

## 1. Introduction

**Figure 1:**
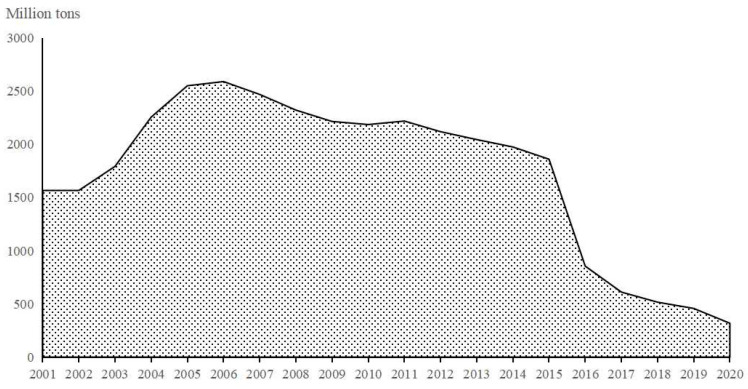
The trend of SO_2_ emissions in China.

Environmental pollution has always been the focus of attention for all sectors of society. Good environmental governance is fundamental for not only the well-being of the region’s population but also for the health of the planet [1]. China has achieved huge economic strides over the past three decades, but the extended development model has also resulted in significant environmental pollution and ecological decline [2], the economic cost of environmental pollution accounts for 8%–15% of China’s annual GDP [3]. According to data on national SO_2_ emissions from 2001 to 2020 published by the Chinese Bureau of Statistics and the Ministry of Environmental Protection, China’s SO_2_ emissions were exceptionally high (Figure 1). To change the traditional extensive economic development model, the “13th Five Year Plan for National Economic and Social Development” was approved in 2015 by the CPC’s 18th Central Committee. The Chinese government has proposed five development concepts: Innovation, coordination, sustainability, openness, and sharing, with an emphasis on the former two. The “Porter hypothesis” contends that coordinating environmental regulation and corporate development through innovation can result in a situation where both parties benefit [4]. Therefore, as far as enterprises are concerned, how to carry out technological innovation while giving consideration to green production is a practical problem that needs to be studied urgently.

China has steadily strengthened its environmental regulations. The “Regulations on the Collection and Use of Pollution Discharge Fees” published in 2003 and the “Environmental Protection Tax Law” published in 2018 both clearly stated that businesses must pay fees for the discharge of pollutants into the environment. Additionally, the local government has published several environmental protection legislative documents to impose fines, control within a period, and halt production for the rectification of businesses’ excessive pollution discharge. These demands from environmental regulations put company managers under pressure to assess the harsh penalties associated with pollution, which has a direct impact on how managers approach environmental regulations. Businesses will accelerate technology innovation to fulfill environmental regulatory standards to avoid paying expensive penalties. To avoid being penalized by environmental supervision, for instance, enhance the industrial process and businesses’ capacity to regulate pollutants and cut emissions. Environmental legislation will encourage businesses to pursue environmentally friendly growth plans [5] as well as to invest more in pollution management [6].

The link between environmental regulation and business innovation is a topic of debate among academics. Enterprise innovation will be hampered in the near term by strict environmental rules that raise production costs and burdens [7,8,9]. However, effective environmental control will have a “compensation for innovation” impact. When the government imposes environmental regulations on businesses, those businesses will enhance their R&D and innovation spending, boost their own levels of invention, and then foster their competitiveness while enhancing environmental quality [10,11,12,13,14].

In contrast to China, western countries have quite diverse environmental regulations. According to the “Porter hypothesis,” when faced with severe environmental regulation measures, businesses will actively improve their technological processes to lessen the impact of regulation costs on the economic performance output of businesses. Furthermore, properly designed environmental regulations are crucial for encouraging technological innovation. As a result, most nations enforce environmental laws like environmental taxes or financial subsidies to repair the ecological environment’s harm. For instance, the United States has passed numerous laws governed by the Environmental Protection Agency; the United Kingdom has imposed high environmental taxes on landfill and air pollution. However, China’s existing market system needs government intervention in the innovative activity incentive process. As a result, China mostly uses the administrative order or government performance assessment system of forced environmental control [15,16]. 

Chinese environmental regulation is directly linked to the political elevation of local government officials within the institutional framework of “political centralization and economic de-centralization” in that country. In the past, the implementation of the official promotion assessment system, which placed a focus on relative economic growth performance, prompted local government officials to hold “promotion tournaments” centered on GDP growth [17]. As a result of the “development before governance” concept, which places human development ahead of environmental protection, local governments often prioritize short-term economic expansion at the cost of resources and the environment. As a result, the formulation and execution of public policies will unavoidably be significantly impacted by the performance orientation and evaluation system design of government employees [18]. China initially made it explicit in 2006 that local cadres’ efficacy in reducing pollution should be considered when making hiring, selection, reward, and punishment decisions. The accountability framework for environmental protection objectives should be rigorously executed, and environmental protection indicators should be incorporated in the official comprehensive assessment and evaluation system as binding indicators [16].

The People’s Republic of China’s Ministry of Environmental Protection signed a letter of responsibility with each province in 2007 regarding the reduction target of the total amount of major pollutants, quantified the reduction target, and divided the emission reduction binding indicators among the provincial governments by signing the letter of responsibility. The provincial governments then divided the emission reduction targets among the municipal governments. Each city also distributes the indications to the main polluting businesses under its control. The accountability system for environmental protection assessment and the “one vote veto” system were added to the Assessment Method for Total Emission Reduction of Major Pollutants published by the State Council of China in 2011, further strengthening the evaluation of local cadres’ performance in emission reduction of pollutants. This demonstrates how local governments with Chinese elements have a distinctive approach to environmental stewardship. Will local officials who are subject to political evaluation pressure and promotion incentives effectively promote environmental governance in their jurisdictions under the green performance assessment system? Does the company have the ability, in accordance with the local government’s appropriate policies, to reduce pollution via technical innovation? In-depth study is really required.

The following are the paper’s contributions: Firstly, it broadens Porter’s hypothesis theory in terms of research theory. This study begins from the standpoint of ex ante regulation of environmental governance goals, in contrast to earlier ex post environmental regulation approaches including pollution levies and government investment in pollution management. Secondly, to the best of its ability, this work has gathered and organized more credible research data at the urban level, strengthening the validity of the study’s findings. According to the literature, most of them are based on provincial government data rather than local government data precisely quantified, which makes it impossible for them to represent the variation more accurately in emission reduction across cities in the province. This article also focuses on the various environmental governance aims of local governments, specifically the variations in governance goals across provinces and between cities at the prefecture level within a province. Finally, in terms of the study sample, this article begins with micro enterprises, compares listed firms’ data to the National Patent Office database, and then explores the influence of local government environmental governance on company technological innovation.

## 2. Theoretical Framework

### 2.1. Environmental Regulation and Enterprise Technology Innovation

Environmental regulations, according to some academics, would drive up business costs and stifle technological innovation [19,20,21,22]. High levels of environmental regulation will lower an organization’s R&D level, and it will be difficult to offset the cost impact with innovation [23]. Research has shown that US environmental legislation has increased the extra expenses faced by US manufacturing companies, decreased their level of competitiveness, and diminished their capacity for technological innovation [11,24,25]. The aggregate number of patent applications filed by German businesses is negatively correlated with the extent of environmental regulatory implementation [26]. Environmental regulations also hinder Chinese businesses’ capacity to innovate technologically [27].

However, it is found that well-crafted environmental control policy instruments may encourage technological advancement and dissemination, leading to a “innovation compensation” impact [28,29,30,31,32,33,34,35,36]. The severity of environmental regulation will be increased, which will encourage the creation of new technologies and environmental R&D initiatives, enhancing economic performance [37,38,39]. Environmental patents are more likely to be issued when environmental expenses are rising [40]. A certain degree of environmental control aids in the development of technological innovation [33,41,42]. It significantly enhances technical innovation as shown by the quantity of patents [43,44,45]. An “inverted U-shaped” link exists between the level of environmental regulation and technological innovation, and the right level of environmental regulation encourages technological change [46]. Environmental control laws, according to some academics, will not encourage technological innovation and dissemination since there is no clear association between environmental regulation and enterprise technology innovation [47]. The quantity of patents and environmental regulating measures do not seem to be related in any clear way [48]. Businesses benefited from investments made in the development of green products and processes [49]. Encourage businesses to take significant, proactive steps to lessen their negative effects on the environment by developing “win–win” solutions [50,51].

### 2.2. Research Assumptions

The Chinese central government and local governments at all levels signed the “Letter of Responsibility for the Reduction of the Total Amount of Major Pollutants in the Eleventh Five Year Plan” in 2007. The emission reduction indicators are divided into provinces, which then divided the indicators into each municipal government, and municipal governments further divided the indicators. In 2011, China’s State Council released the “Assessment Method for Total Emission Reduction of Major Pollutants,” which strengthened the evaluation of local government officials’ environmental performance in pollutant emission reduction by adding the accountability system for environmental protection assessment and the “one-vote veto” system. The regional, industrial, and enterprise disparities were taken into consideration by the local governments as they promoted the reduction of pollutant emissions. To encourage balanced growth of all areas, they made varied changes to the emission reduction objectives rather than using a “one size fits all” approach.

The local government will implement stronger environmental control rules to encourage businesses to perform green production and R&D innovation via the “Anti-driving impact” to meet the environmental protection assessment targets established by the superior government [52]. According to the “reverse force effect” of Porter’s hypothesis [53], businesses were forced to incorporate green production and technological innovation into their business decisions and strategic planning because of the local government’s stringent environmental regulations. This helped businesses achieve a “win–win” situation where environmental protection and business competitiveness were both enhanced. The following assumptions are suggested by this research based on the data presented above:

**Hypothesis** **1.** *Local governments have developed regionally distinct environmental governance goals. Enterprise technology innovation levels will be increasingly important as environmental governance goals become more stringent.*

If a company’s technological innovation operations are exclusively reliant on the market, they will not be able to provide effective innovation incentives due to their long cycle, expensive investment, and high risk [54]. The government can enact a range of incentive policies, such as financial incentives, environmental protection subsidies, tax breaks, etc., through the “compensation effect” and “incentive effect” to encourage businesses to carry out technological innovation, provide funding sources and convenient conditions, alleviate the lack of R&D funds, and reduce the cost of enterprise innovation [55]. The government’s financial subsidy is a clear financial policy that might significantly increase an enterprise’s cash flow revenues. It may also significantly alleviate organizations’ concerns about the unpredictable nature of innovation activities and boost their enthusiasm for technological innovation, supporting technological innovation and innovation effectiveness. Based on the information provided, this study suggests the following hypotheses:

**Hypothesis** **2.** *Local government environmental governance can promote the technological innovation level of enterprises through financial subsidies.*

## 3. Literature Review

### 3.1. Environmental Governance and Green Performance Assessment

Financial independence and discretion are granted to local governments in topics falling within their purview. The official tenure evaluation system is used by the central government to monitor and encourage local representatives. In addition to caring about the welfare of the local populace and regional growth, local politicians also have their political careers in mind. They engage in heated competition for a small number of promotion positions. It is thought of as a “promotion championship” throughout this procedure [17]. To optimize their tenure performance, local authorities will modify their own goal function in accordance with the performance appraisal’s content [56]. Local authorities will alter their competing tactics in response to the “promotion tournament,” dismissing signs that are beyond the purview of the evaluation and concentrating primarily on those that are evaluated. Therefore, one of the internal motives for municipal authorities to actively support environmental governance is political promotion.

The nexus between environmental performance evaluation and government actions, environmental governance, and economic factors remained the subject of previous studies. The environmental evaluation that focuses on resource conservation and environmental improvement has a beneficial impact on the promotion of officials because of the assessment system’s variety [57]. Corporate environmental governance is subject to periodic change because of the environmental performance assessment [58]; the impact of the environmental performance assessment on local governments is also reflected in the creation and implementation of environmental governance goals and policies [59]. The presence of political incentives means that the conduct of the government is immediately impacted by the environmental performance evaluation of the top cadres [60]. For instance, introducing and putting into practice environmental rules like environmental objective restrictions may aid in promoting industrial transformation and improvement [61].

Local governments in China compete for funding for environmental governance initiatives, and “race to the bottom” traits are increasingly pronounced [62]. The association between environmental degradation and local politicians’ promotion is moderated by the campaign-style governing practices of local authorities [63]. Indeed, local governments experience a “race to the top” as a result of environmental restrictions [64].

### 3.2. Impact of Heterogeneous Environmental Regulation on Enterprise Technological Innovation

The technological innovation of businesses has been significantly aided by five different environmental regulation policies (tax, technical and non-technical emission standards, market mechanisms, and government subsidies) [65], with technical emission standards having the least incentive effect. The greatest positive stimulating impact of pollutant discharge regulations and levies on environmental research and development is under Cournot’s competitive market structure [66]. While voluntary environmental regulation laws have a large beneficial influence, the positive link between command-and-control environmental regulation and technological innovation is not significant [67]. More so than command and control environmental regulation, market incentives for environmental regulation may promote the development of green technologies [68,69,70].

The potential for technological innovation and the enterprise’s emissions are connected in terms of how various environmental control policy instruments affect endogenous technological innovation [71]. Mandatory environmental control laws and the development of corporate environmental technologies are clearly related. Enterprise technology innovation is unaffected by lax obligatory environmental control regulations (such as corporate social responsibility disclosure rules) [72]. It is discovered that increasing the pollutant discharge tax rate and pollutant discharge license fee may boost the green technology innovation of firms [73] by comparing and assessing the effects of various environmental control measures on the green technology innovation of enterprises. The largest impact of emission trading licenses is the technological incentive effect [74,75].

To sum up, in the existing literature on the relationship between environmental issues and enterprise innovation, it is found that most of the previous studies focused on different environmental regulation intensity and how heterogeneous environmental regulation tools affect innovation activities, while few studies discussed the responsiveness of enterprises to this policy from the perspective of prior regulation of different environmental governance goals. When analyzing the innovation incentive effect of environmental regulation, the samples of existing research mainly focus on industry or regional panel data, which cannot start from the main body of technological innovation, enterprises, and lack of empirical evidence at the micro enterprise level. Therefore, from the perspective of green performance assessment of local governments with Chinese characteristics, this paper examines how local government officials, under the dual pressure of environmental governance goals and environmental performance assessment, can achieve “win–win” between enterprise technology innovation and environmental protection.

## 4. Material and Methods

### 4.1. Empirical Framework 

To discuss the impact of local government environmental governance objectives on enterprise technological innovation, this paper establishes a fixed effect model for research, and constructs the following econometric model based on the above theoretical analysis:

(1) $$ETI_{\mu ,i,t}=\alpha_{0}+\alpha_{1}\mathrm{Governanc}e_{i,t}\times D_{i}+\alpha_{n}\mathrm{Controls}+f_{\mu }+f_{i}+f_{t}+\varepsilon_{i,t}$$

where μ represents the listed company, if represents the city, and t represents the year. ${\mathrm{ETI}}_{\mu ,i,t}$ represents the number of invention patent applications and authorizations of listed companies μ in city i in year t and takes the number of invention patent applications and authorizations plus 1 as the proxy variable of the enterprise’s technological innovation level. ${\mathrm{Governance}}_{i,t}$ is the core explanatory variable, namely the environmental governance goals of local government, and the pollutant ${\mathrm{SO}}_{2}$ emission reduction target is used as the proxy variable. $D_{i}$ is a dummy variable of ${\mathrm{SO}}_{2}$ key emission industries. When the enterprise μ belongs to a key emission industry, the value is 1, otherwise it is 0. In addition, Controls are a series of other factors that affect the technological innovation of enterprises. $f_{\mu }$ refers to individual fixed effect, $f_{i}$ refers to city fixed effect, $f_{t}$ is year fixed effect and $\varepsilon_{i,t}$ is random error term, which is assumed to be normally distributed at zero means value and constant variance [76,77,78]. 

Governments at all levels have taken into account the differences in resource endowments, technological innovation, and management levels among regions, industries, and enterprises to achieve the “win–win” of economic growth and environmental protection and health. Instead of adopting the “one size fits all” model, they have set different environmental governance goals to encourage balanced development in all regions [79,80,81]. For instance, to compel local governments to optimize and improve the industrial structure and implement green innovation, developed regions like those on the east coast are obliged to accomplish higher environmental target assessment tasks. The government will suitably lower its environmental protection assessment objectives in the central and western regions to support the fast economic growth of those areas owing to their poor economic foundations and the effect of regional factors. The national emission objective for Shanghai is to cut emissions by 26 percent, whereas Heilongjiang Province’s responsibility in the center and western provinces is just 3.52 percent, to use the “11th Five Year Plan” as an example. Cities within the same province vary as well. Jiangsu Province mandates a 53.6 percent reduction for Xuzhou City, but just a 2.8 percent reduction for Suqian City. The superior government will design the environmental protection assessment targets differently based on the variations in the economic development levels of each area.

Therefore, this paper focuses on the impact of the difference of environmental governance objectives between provinces and prefecture level cities within their jurisdiction on enterprise innovation behavior, so this paper constructs the following measurement model:

(2) $$\mathrm{ET}I_{\mu ,i,t}=\alpha_{0}+\alpha_{1}\mathrm{DER}T_{SO_{2}}\times D_{i}+\beta_{n}\;\mathrm{Controls}+\;f_{\mu }+f_{i}+f_{t}+\varepsilon_{i,t}$$

where μ represents the listed company, i represents the city, and t represents the year. ${\mathrm{ETI}}_{\mu ,i,t}$ represents the enterprise’s technological innovation level. The setting method is consistent with the model (1). ${\mathrm{DERT}}_{{\mathrm{SO}}_{2}}$ refers to the difference between the ${\mathrm{SO}}_{2}$ emission reduction targets of each province and each prefecture level city within its jurisdiction, which represents the differentiated environmental governance goals of the local government. $D_{i}$ is a dummy variable of ${\mathrm{SO}}_{2}$ key emission industries. When the enterprise μ belongs to a key emission industry, the value is 1, otherwise it is 0. In addition, Controls are a series of other factors that affect the technological innovation of enterprises. $f_{\mu }$ refers to individual fixed effect, $f_{i}$ refers to city fixed effect, $f_{t}$ is year fixed effect and $\varepsilon_{i,t}$ is random error term.

In addition, China’s A-share listed companies involve a wide range of industries, and not all listed companies are involved in the emission of ${\mathrm{SO}}_{2}$. Referring to the practice of existing literature, and according to the First National Pollution Source Census Bulletin, we distinguish between ${\mathrm{SO}}_{2}$ key emission industries and non ${\mathrm{SO}}_{2}$ key emission industries [75,82]. The electric power industry, non-metallic mineral products industry, non-ferrous metal smelting and processing industry, chemical raw materials and chemical products manufacturing industry, petroleum processing and coking and nuclear fuel processing industry are classified as ${\mathrm{SO}}_{2}$ key emission industries.

Additionally, a variety of sectors are represented by China’s A-share listed businesses, and not all of them are engaged in SO_2_ emission. We make a distinction between SO_2_ key emission sectors and non SO_2_ key emission businesses using the conventions of the body of current research and the First National Pollution Source Census Bulletin [75,82]. The following industries are categorized as SO_2_ main emission industries: electric power generation, non-metallic mineral products, non-ferrous metal smelting and processing, chemical raw material and chemical product production, petroleum processing and coking, and nuclear fuel processing.

### 4.2. Definitions of Variables

#### 4.2.1. Explained Variable: Enterprise Technological Innovation

This study follows the previous literature to measures the technological innovation capability of enterprises by the number of invention patent applications and authorizations [83,84,85,86]. The number of invention patent applications measures the number of enterprises’ innovation, while the number of invention patent authorizations measures the quality of enterprises’ innovation.

#### 4.2.2. Core Explanatory Variable

This paper collates the emission reduction target data of 230 cities from 2006 to 2019 to measure the environmental governance goals of local governments. The differential environmental governance goals are measured by the difference between the emission reduction goals of each city and its province.

#### 4.2.3. Control Variables

Referring to the existing research, at the enterprise level, we selected the enterprise size, enterprise age, asset liability ratio, fixed asset ratio, and total asset yield as the control variables. The urban level, urban population, and economic development level were selected as control variables [87].

### 4.3. Data Source and Processing

We select the data of invention patent applications and authorizations of A-share listed companies in China from 2006 to 2019 and the corresponding economic data of enterprises, industries, and cities to empirically test the impact of local government environmental governance objectives on enterprise technology innovation. Among them, the technical innovation data comes from the patent retrieval and incoPat global patent database of the State Patent and Property Office (SIPO). We also used the “International Patent Classification List” of the World Intellectual Property Organization (WIPO) for conditions [83,88] to identify the number of invention patent applications and authorizations of A-share listed companies. The WIND database and the China Research and Development (CNRDS) database were utilized to provide the business characteristics and financial data for this article. The information on each province’s and city’s pollutant emission reduction goal collected from the relevant policy papers of the federal, provincial, and local government agencies. From 2006 to 2019, the China Urban Statistical Yearbook and the China Statistical Yearbook were used to compile economic and financial statistics for 230 cities. In this study, the samples with incomplete data are removed to increase the data’s representativeness.

## 5. Results and Discussion

### 5.1. Benchmark Regression 

The results of the benchmark regression results of model (1) are given in Table 1. Under the fixed effects of city, year and individual, the environmental governance objectives of local governments have a significant role in promoting the quantity and quality of enterprise technological innovation. In terms of the number of innovations, the estimated coefficient of the environmental governance goal is significantly positive at the level of 1%. On this basis, a series of control variables are further added to column (2), and the impact of environmental governance objectives on enterprise technological innovation is still very stable, which also passed the 1% significance test. In terms of innovation quality, the estimated coefficient of environmental governance goals is significantly positive at the level of 5%. The findings demonstrated that, regardless of the amount or quality of innovation, firms’ technical innovation level has greatly increased in response to the local government’s high intensity environmental governance aims. 

### 5.2. Differential Environmental Governance Goals and Enterprise Technological Innovation

This section primarily looks at how varied environmental governance goals across provinces and cities within the province affect the development of business technology. The benchmark regression results of model (2) are shown in Table 2. From Column (1) and Column (3), the gap between the environmental governance goals of provinces and cities has significantly promoted the technological innovation of enterprises, and the estimated coefficient of ${\mathrm{DERT}}_{{\mathrm{SO}}_{2}}$ is statistically significantly positive at the level of 1% and 5%. After adding a series of control variables in Column (2) and Column (4), they all passed the significance test, and the results are still very robust. It shows that local governments have different environmental governance objectives, and the differential distribution of such environmental governance objectives further strengthens the technological innovation of enterprises (Hypothesis 1 is validated).

### 5.3. Robustness Test

This section performs a heterogeneity test on the ownership of the company, the area where the firm is situated, and the size of the enterprise since these three factors may have an influence on how diverse environmental governance goals affect enterprise technical innovation.

#### 5.3.1. Enterprises with Different Ownership

The incentive effect of the policy varies with enterprise characteristics and regional characteristics: The green innovation effect of the policy is more obvious in large-sized and state-owned companies [89]. The ownership attribute of an enterprise usually has different effects on its R&D investment and technological innovation. This paper divides the enterprises in the selected sample into two groups: State-owned enterprises and non-state-owned enterprises for the heterogeneity test to verify the correctness of the benchmark regression results. Table 3 reports the heterogeneous impact of different environmental governance objectives on the technological innovation of state-owned enterprises and non-state-owned enterprises. The results show that both the quantity and quality of technological innovation in non-state-owned enterprises have a positive impact, while for state-owned enterprises, there is no significant impact. The reason is that the state-owned enterprises have a great impact on the local economic development, have enjoyed the policy support of the local government, including financial subsidies, tax relief and other aspects [90,91], and are not sensitive to various environmental regulations. In contrast, most non-state-owned enterprises are private enterprises. Under the pressure of high-level environmental regulation, they need to adjust the R&D direction in time according to the policy guidance. Therefore, non-state-owned enterprises usually show higher innovation enthusiasm and flexibility. This is like the conclusion of previous studies [82].

The policy’s incentive impact varies according to firm characteristics and geographical features; it is particularly pronounced in big, state-owned businesses [89]. An enterprise’s ownership characteristic often has a variety of impacts on its R&D spending and technical innovation. To perform a heterogeneity test and ensure that the findings of the benchmark regression are accurate, this study splits the businesses in the chosen sample into two groups: state-owned businesses and non-state-owned businesses. Table 3 shows the varied effects of various environmental governance goals on technological innovation by state-owned and non-state-owned businesses. The findings indicate that although there is no discernible influence for state-owned organizations, there is a positive correlation between the amount and quality of technical innovation in non-state-owned businesses. The explanation is that state-owned businesses have a significant influence on local economic growth, have benefited from local government policy assistance in the form of cash incentives, tax breaks, and other benefits, and are less susceptible to different environmental regulations [90,91]. In contrast, private businesses make up most non-state-owned businesses. High-level environmental regulations are putting pressure on them, so they must quickly change the course of their R&D in accordance with the policy recommendations. As a result, non-state-owned businesses often exhibit more creative zeal and adaptability. These results are according to the findings of a previous study [82].

#### 5.3.2. Enterprises in Different Regions

There are large differences in the economic development level of different regions in China. Therefore, to investigate the regional differences in the impact of environmental governance objectives on enterprise technological innovation, this paper divides 230 prefecture level cities into the eastern region and the central and western regions by region and conducts a regional heterogeneity test to verify the robustness of the research conclusions. The influence of environmental governance goals on the quantity and caliber of innovations made by firms in the eastern area is notably positive at the level of 1% and 5%, as shown in Table 4. The results found that there is little or no effect of environmental governance goals on technical innovation of businesses in the central and western areas. The explanation is that the central and western regions’ economies are weak, and their industrial structures are archaic. Enterprises’ ability to innovate technologically is somewhat hampered by the higher environmental governance aims of the government since they are forced to halt work, manufacturing, and other activities in order to comply with the government’s environmental protection regulations.

#### 5.3.3. Enterprises of Different Scales 

The findings in Table 5 detail the effects of environmental governance goals on technological innovation by businesses of various sizes. From columns (1) to (4), the promotion effect of environmental governance goals on the quantity of innovation in large enterprises is significantly positive at the levels of 1% and 5%, and the impact of various environmental goals between provinces and cities on the quality of innovation in large enterprises is significantly positive at the level of 5%. Furthermore, the results confirmed that SMEs have little effect on technical innovation. Large businesses may reap higher rewards more quickly since they have amassed more innovation resources. Major ecosystems are also increasingly driving the growth of businesses, and these ecosystems must be effectively managed by powerful and large businesses. Therefore, skewed emission reduction goal limits encourage big firms to innovate in technology more.

### 5.4. Robustness Test

To verify the reliability of the estimation results, this study uses the number of green invention patents, utility model patents and appearance design patents of listed companies to measure the innovation output level of enterprises. At the same time, the natural logarithm of per capita R&D expenditure of enterprises is used to measure the innovation investment level of enterprises [92]. Table 6 reports the regression results after changing the explained variables. Whether from the perspective of innovation input or innovation output, environmental governance objectives have a significant role in promoting enterprise technological innovation, and the above results are consistent with the conclusions of this paper.

### 5.5. Problem of Endogeneity 

While it is disproved that the urban heterogeneity and systematic changes in macro factors have an unconvincing impact on the conclusions of this paper, it is still possible that there is a reverse causal relationship between the constraint intensity of local biased emission reduction targets and enterprise technological innovation, which would cause some deviation to the conclusions of this paper.

Finding the right tool variables is required to address the model’s endogenous issue. Using the techniques from previous study, the urban air flow coefficient is chosen as the tool variable in this article [93,94]. The monitoring concentration of key pollutants will be greater the smaller the air flow coefficient. To increase the level of environmental goals’ restraint, the local government will implement tougher environmental monitoring methods. The air flow coefficient satisfies the exogenous criteria of tool variables since it is dependent on the local wind speed and other natural elements and is unaffected by the economic activity of prefecture level cities. The tool variables’ regression results are shown in Table 7. The statistics in the first stage are all bigger than the crucial value of the audited F value at the 10% error level, showing that there is no issue with weak tool variables from the standpoint of the efficacy of tool variables. The computed coefficient met the 1% threshold for significance, and its sign was consistent with the fundamental regression findings.

### 5.6. Mechanism Analysis 

Based on the theoretical analysis and empirical results, in recent years, under the institutional mechanism of “upward responsibility” of local officials, the task of pollutant emission reduction has been broken down to local governments from top to bottom, giving local officials greater pressure on environmental regulation. Local government officials pay more and more attention to environmental performance in the assessment and evaluation system. Enterprises may face greater external pressure on environmental protection in order to meet the requirements of the government. For example, tax rebates have a significant incentive effect on enterprise technological innovation and do not exert pressure on firms. Therefore, its anti-driving effect on firm technological innovation is not obvious [95]. However, resource tax can improve the technological innovation of mining firms through anti-driving and incentive effects, thereby promoting the coordinated development of the economy and the environment [96]. In the face of greater environmental regulatory pressure, local governments will grant environmental subsidies to enterprises and require enterprises to take the initiative to protect the environment [97,98]. After receiving the environmental protection subsidies from the government, enterprises will also meet the environmental protection requirements of local governments and more enthusiastically use more funds for technological innovation [99,100,101]. The given results in Table 8 reported that the intermediate mechanism between the local government’s environmental governance goals and environmental protection subsidies. There is a positive correlation with environmental protection subsidies at the 10% significance level. This shows that the larger the emission reduction target allocated by the superior government, the more environmental protection subsidies the local government will give to enterprises to stimulate technological innovation. Therefore, the environmental governance of local governments has been effectively verified through positive incentives to promote enterprise technological innovation. The results validated the Hypothesis 2.

## 6. Conclusion, Policy Implications, and Limitations of the Study

### 6.1. Conclusion and Policy Implications

The government should strengthen the restriction of environmental governance objectives and promote the technological innovation and industrial structure upgrading of enterprises, which are the inherent requirements for achieving high-quality economic development. Therefore, from the perspective of the green performance assessment of local governments with Chinese characteristics, this study focuses on the impact of local government environmental governance goals on enterprise technology innovation by sorting out pollutant emission reduction target data of 230 cities in China from 2006 to 2019. According to the findings, there are clear regional disparities in the environmental governance goals set out by the local government, which are shown as lower governance indicators for undeveloped regions and higher governance indicators for developed areas. The environmental governance goals have a more positive influence on technological innovation in businesses in developed areas, big businesses, and private businesses. The results of a mechanism analysis demonstrated that the local governments favored the use of financial incentives to raise businesses’ levels of technical innovation. The study’s findings provide a realistic foundation for achieving the “win–win” of excellent green economic growth and local government environmental stewardship.

- Further improvement the performance evaluation dimensions, and rating system of local government officials is required. Efforts are required to increase the proportion of environmental protection and ecological governance in the assessment level system, implement the lifelong responsibility system for environmental protection, and effectively regulate the environmental pollution caused by local governments’ pursuit of economic development
- It is required to consider the heterogeneous impact of regional differences and to formulate environmental regulation policies that are in line with reality and local conditions. There are differences between the eastern coastal areas and the central and western regions in terms of economic development level, resource endowment, industrial layout, pollution level, etc. Therefore, the superior government should be targeted when implementing high-level environmental regulation policies, fully consider regional differences, combine with local actual conditions, and strictly prohibit “one size fits all” environmental regulation policies.
- Local governments should strengthen the screening and supervision of subsidized enterprises, establish a reasonable evaluation mechanism, and formulate targeted innovation subsidy policies for corresponding types of enterprises.

### 6.2. Limitations of the Study

This study focuses on the green performance assessment and enterprise technology innovation with Chinese characteristics, with less comparative analysis with other countries. Subsequent studies should focus on data collection from other countries for comparative analysis. Using SO_2_ emission reduction target data as proxy variables to measure the environmental governance goals of local governments has certain limitations. Moreover, the current study involves the analysis of differences between different regions. For example, the increase in the intensity of environmental regulation will have an impact on the government behavior of surrounding cities, thus producing spillover effects on green technology innovation. Therefore, further research can establish a spatial econometric model for empirical analysis.

## Figures and Tables

**Table 1:** Results of benchmark regression.

Variables	ETI			
	Number of Innovations		Quality of Innovation	
	(1)	(2)	(3)	(4)
Governance	0.085 ***	0.079 ***	0.045 **	0.046 **
	(0.020)	(0.019)	(0.020)	(0.021)
Size		0.029		0.037 *
		(0.026)		(0.020)
Age		0.174 ***		0.219 ***
		(0.054)		(0.039)
Lev		0.033		0.152 **
		(0.101)		(0.065)
Tangibility		0.112 ***		0.350 ***
		(0.087)		(0.084)
Roa		−0.033 **		−0.072 **
		(0.014)		(0.028)
Agdp		0.108		0.007
		(0.086)		(0.076)
Population		0.002		0.035
		(0.071)		(0.048)
Cons	0.685 ***	−3.584 ***	0.559 ***	−4.848 ***
	(0.001)	(1.211)	(0.001)	(0.986)
City-level FE	Yes	Yes	Yes	Yes
Year FE	Yes	Yes	Yes	Yes
Individual FE	Yes	Yes	Yes	Yes
*N*	26,288	26,288	26,288	26,288
*R* ^2^	0.727	0.728	0.727	0.728

Standard errors are given in parentheses. ***, ** and * represent the level of significance of parameters at 1%, 5%, and 10%, respectively.

**Table 2:** Empirical results of different environmental governance goals.

Variables	ETI			
	Number of Innovations		Quality of Innovation	
	(1)	(2)	(3)	(4)
${\mathrm{DERT}}_{{\mathrm{SO}}_{2}}$	0.072 ***	0.066 ***	0.045 **	0.043 **
	(0.020)	(0.020)	(0.018)	(0.018)
Size		0.029		0.037 *
		(0.026)		(0.020)
Age		0.174 ***		0.219 ***
		(0.054)		(0.040)
Lev		0.033		0.152 **
		(0.101)		(0.065)
Tangibility		0.112 ***		0.350 ***
		(0.087)		(0.084)
Roa		−0.033 **		−0.072 **
		(0.014)		(0.028)
Agdp		0.109		0.007
		(0.087)		(0.076)
Population		0.001		0.035
		(0.071)		(0.048)
Cons	0.689 ***	−4.471 ***	0.561 ***	−4.848 ***
	(0.000)	(1.575)	(0.000)	(0.988)
City-level FE	Yes	Yes	Yes	Yes
Year FE	Yes	Yes	Yes	Yes
Individual FE	Yes	Yes	Yes	Yes
*N*	26,288	26,288	26,288	26,288
*R* ^2^	0.727	0.730	0.728	0.730

Standard errors are given in parentheses. ***, ** and * represent the level of significance of parameters at 1%, 5%, and 10%, respectively.

**Table 3:** Empirical results of the heterogeneity analysis of enterprises with different ownership.

Variables	State-Owned Enterprise				Non-State-Owned Enterprises			
	Number of Innovations		Quality of Innovation		Number of Innovations		Quality of Innovation	
	(1)	(2)	(3)	(4)	(5)	(6)	(7)	(8)
Governance	0.172		0.196		0.077 ***		0.038 ***	
	(0.145)		(0.158)		(0.014)		(0.014)	
${\mathrm{DERT}}_{{\mathrm{SO}}_{2}}$		0.004		0.176		0.082 ***		0.040 ***
		(0.176)		(0.181)		(0.011)		(0.010)
Cons	−5.113 **	−5.128 **	−1.123	−1.126	−6.463 ***	−6.452 ***	−0.685	−0.687
	(2.129)	(2.127)	(1.289)	(1.289)	(1.368)	(1.372)	(0.948)	(0.948)
Control variable	Yes	Yes	Yes	Yes	Yes	Yes	Yes	Yes
City-level FE	Yes	Yes	Yes	Yes	Yes	Yes	Yes	Yes
Year FE	Yes	Yes	Yes	Yes	Yes	Yes	Yes	Yes
Individual FE	Yes	Yes	Yes	Yes	Yes	Yes	Yes	Yes
*N*	11,177	11,177	11,177	11,177	14,656	14,656	14,656	14,656
*R* ^2^	0.761	0.761	0.691	0.691	0.76	0.76	0.701	0.701

Standard errors are given in parentheses. *** and ** represent the level of significance of parameters at 1%, and 5%, respectively.

**Table 4:** Heterogeneity analysis of enterprises in various regions.

Variables	Enterprises in Eastern Regions				Enterprises in Central and Western Regions			
	Number of Innovations		Quality of Innovation		Number of Innovations		Quality of Innovation	
	(1)	(2)	(3)	(4)	(5)	(6)	(7)	(8)
Governance	0.076 ***		0.053 **		0.115		−0.080	
	(0.013)		(0.023)		(0.230)		(0.144)	
${\mathrm{DERT}}_{{\mathrm{SO}}_{2}}$		0.065 ***		0.041 ***		0.162		0.040
		(0.018)		(0.015)		(0.300)		(0.196)
Cons	−2.187	−2.194	−4.657 ***	−4.670 ***	−0.926	−0.9	0.359	0.332
	(1.982)	(1.992)	(1.542)	(1.558)	(1.222)	(1.231)	(1.096)	(1.099)
Control variable	Yes	Yes	Yes	Yes	Yes	Yes	Yes	Yes
City-level FE	Yes	Yes	Yes	Yes	Yes	Yes	Yes	Yes
Year FE	Yes	Yes	Yes	Yes	Yes	Yes	Yes	Yes
Individual FE	Yes	Yes	Yes	Yes	Yes	Yes	Yes	Yes
*N*	18,219	18,219	18,219	18,219	8028	8028	8028	8028
*R* ^2^	0.741	0.741	0.745	0.745	0.699	0.699	0.691	0.691

Standard errors are given in parentheses. *** and ** represent the level of significance of parameters at 1%, and 5%, respectively.

**Table 5:** Heterogeneity Analysis of Enterprises of Different Sizes.

Variables	Large Enterprises				Small and Medium Enterprises			
	Number of Innovations		Quality of Innovation		Number of Innovations		Quality of Innovation	
	(1)	(2)	(3)	(4)	(5)	(6)	(7)	(8)
Governance	0.061 ***		0.050 **		0.11		0.069	
	(0.023)		(0.020)		(0.104)		(0.091)	
${\mathrm{DERT}}_{{\mathrm{SO}}_{2}}$		0.048 **		0.054 ***		0.269		−0.048
		(0.023)		(0.016)		(0.241)		(0.229)
Cons	−5.774 ***	−5.779 ***	−6.157 ***	−6.147 ***	−0.938	−0.918	−0.463	−0.464
	(1.916)	(1.914)	(1.294)	(1.297)	(0.773)	(0.775)	(0.795)	(0.793)
Control variable	Yes	Yes	Yes	Yes	Yes	Yes	Yes	Yes
City-level FE	Yes	Yes	Yes	Yes	Yes	Yes	Yes	Yes
Year FE	Yes	Yes	Yes	Yes	Yes	Yes	Yes	Yes
Individual FE	Yes	Yes	Yes	Yes	Yes	Yes	Yes	Yes
*N*	20,862	20,862	20,862	20,862	5299	5299	5299	5299
*R* ^2^	0.733	0.732	0.742	0.742	0.729	0.729	0.688	0.688

Standard errors are given in parentheses. *** and ** represent the level of significance of parameters at 1%, and 5%, respectively.

**Table 6:** Robustness test: Changing the agent variables of enterprise technological innovation.

Variables	Green Invention Patent		Utility Model Patent		Industrial Design Patent		R&D Expenditure	
	(1)	(2)	(3)	(4)	(5)	(6)	(7)	(8)
Governance	0.068 ***		0.065 **		0.044 ***		0.176 ***	
	(0.013)		(0.031)		(0.011)		(0.054)	
${\mathrm{DERT}}_{{\mathrm{SO}}_{2}}$		0.074 ***		0.045 **		0.044 ***		0.148 ***
		(0.009)		(0.018)		(0.008)		(0.046)
Cons	−2.015 ***	−2.005 ***	−2.341 *	−2.354 *	0.202	0.205	7.197 ***	7.180 ***
	(0.444)	(0.445)	(1.236)	(1.234)	(0.589)	(0.589)	(1.467)	(1.468)
Control variable	Yes	Yes	Yes	Yes	Yes	Yes	Yes	Yes
City-level FE	Yes	Yes	Yes	Yes	Yes	Yes	Yes	Yes
Year FE	Yes	Yes	Yes	Yes	Yes	Yes	Yes	Yes
Individual FE	Yes	Yes	Yes	Yes	Yes	Yes	Yes	Yes
*N*	25,362	25,362	26,288	26,288	26,288	26,288	15,222	15,222
*R* ^2^	0.621	0.621	0.693	0.693	0.662	0.662	0.785	0.785

Standard errors are given in parentheses. ***, ** and * represent the level of significance of parameters at 1%, 5%, and 10%, respectively.

**Table 7:** Regression results of tool variables.

Variables	Number of Innovations		Quality of Innovation	
	(1)	(2)	(3)	(4)
Governance	2.933 ***	3.086 **	1.731 *	1.995 *
	(1.031)	(1.262)	(0.944)	(1.121)
Size		0.040		0.017
		(0.035)		(0.026)
Age		0.124 **		0.210 ***
		(0.057)		(0.044)
Lev		0.001		0.11
		(0.088)		(0.070)
Tangibility		0.340 ***		0.326 ***
		(0.128)		(0.096)
Roa		−0.018		−0.059 ***
		(0.014)		(0.015)
Agdp		−0.060		−0.073
		(0.111)		(0.083)
Population		0.009		0.030
		(0.035)		(0.026)
City-level FE	Yes	Yes	Yes	Yes
Year FE	Yes	Yes	Yes	Yes
Individual FE	Yes	Yes	Yes	Yes
Regression results in the first stage				
IV	0.000 ***	0.000 ***	0.000 ***	0.000 ***
	(0.000)	(0.000)	(0.000)	(0.000)
Control variable	Yes	Yes	Yes	Yes
City-level FE	Yes	Yes	Yes	Yes
Year FE	Yes	Yes	Yes	Yes
Individual FE	Yes	Yes	Yes	Yes
First stage F-value	20.8 ***	12.75 ***	20.78 ***	12.74 ***
*N*	29,077	28,923	29,076	28,922

Standard errors are given in parentheses. ***, ** and * represent the level of significance of parameters at 1%, 5%, and 10%, respectively.

**Table 8:** Empirical results of environmental subsidies.

Variables	Environmental Subsidies	
	(1)	(2)
Governance	0.844 *	
	(0.461)	
${\mathrm{DERT}}_{{\mathrm{SO}}_{2}}$		0.999 *
		(0.561)
Cons	4.713 **	4.930 **
	(2.172)	(2.147)
Control variable	Yes	Yes
City-level FE	Yes	Yes
Year FE	Yes	Yes
Industry FE	Yes	Yes
*N*	2521	2521
*R* ^2^	0.314	0.314

Standard errors are given in parentheses. ** and * represent the level of significance of parameters at 5%, and 10%, respectively.

## Data Availability

The data used to support the findings of this study are available from the corresponding author upon request.
